# Polysaccharide-Based Bilayer Coatings for Biofilm-Inhibiting Surfaces of Medical Devices

**DOI:** 10.3390/ma14164720

**Published:** 2021-08-21

**Authors:** Urban Ajdnik, Thomas Luxbacher, Alenka Vesel, Alja Štern, Bojana Žegura, Janja Trček, Lidija Fras Zemljič

**Affiliations:** 1 Institute of Engineering Materials and Design, Faculty of Mechanical Engineering, University of Maribor, Smetanova 17, 2000 Maribor, Slovenia; 2 Anton Paar GmbH, Anton Paar Strasse 20, 8054 Graz, Austria; thomas.luxbacher@anton-paar.com; 3 Department of Surface Engineering and Optoelectronics, Jožef Stefan Institute, Teslova 30, 1000 Ljubljana, Slovenia; alenka.vesel@ijs.si; 4 Department of Genetic Toxicology and Cancer Biology, National Institute of Biology, Večna Pot 111, 1000 Ljubljana, Slovenia; Alja.Stern@nib.si (A.Š.); Bojana.Zegura@nib.si (B.Ž.); 5 Department of Biology, Faculty of Natural Sciences and Mathematics, University of Maribor, Koroška cesta 160, 2000 Maribor, Slovenia; janja.trcek@um.si

**Keywords:** polysaccharides, chitosan, hyaluronic acid, anti-biofilm, silicone

## Abstract

Chitosan (Chi) and 77KS, a lysine-derived surfactant, form polyelectrolyte complexes that reverse their charge from positive to negative at higher 77KS concentrations, forming aggregates that have been embedded with amoxicillin (AMOX). Dispersion of this complex was used to coat polydimethylsiloxane (PDMS) films, with an additional layer of anionic and hydrophilic hyaluronic acid (HA) as an outer adsorbate layer to enhance protein repulsion in addition to antimicrobial activity by forming a highly hydrated layer in combination with steric hindrance. The formed polysaccharide-based bilayer on PDMS was analyzed by water contact angle measurements, X-ray photoelectron spectroscopy (XPS), and surface zeta (*ζ*)-potential. All measurements show the existence and adhesion of the two layers on the PDMS surface. Part of this study was devoted to understanding the underlying protein adsorption phenomena and identifying the mechanisms associated with biofouling. Thus, the adsorption of a mixed-protein solution (bovine serum albumin, fibrinogen, γ-globulin) on PDMS surfaces was studied to test the antifouling properties. The adsorption experiments were performed using a quartz crystal microbalance with dissipation monitoring (QCM-D) and showed improved antifouling properties by these polysaccharide-based bilayer coatings compared to a reference or for only one layer, i.e., the complex. This proves the benefit of a second hyaluronic acid layer. Microbiological and biocompatibility tests were also performed on real samples, i.e., silicone discs, showing the perspective of the prepared bilayer coating for medical devices such as prostheses, catheters (balloon angioplasty, intravascular), delivery systems (sheaths, implants), and stents.

## 1. Introduction

Nowadays, an effective implant material should have a functional surface that is antimicrobial, biocompatible with the human organism and disinfects the surrounding tissue after insertion without affecting the human body’s immune response to microorganisms. Therefore, current research focuses on advanced surface treatments that prevent microorganism adhesion, inhibit their growth, and interrupt biofilm formation [1,2]. Surface treatment with polyelectrolytes is one of the promising means to combat implant-associated biofilm infections [3]. Polyelectrolytes are charged polymers consisting of ionizable groups attached to polymer chains and show a strong tendency to adsorb on solid surfaces. Accordingly, they are valued as surface coatings and can be used in many processes, including antifouling coatings to prevent protein adsorption and biofilm formation [4] and especially as bioactive coatings for medical devices [5,6]. Chitosan (Chi), a naturally abundant polymer offers excellent biodegradability, biocompatibility, and processability while being safe for human health. The polycationic nature of Chi exhibits antimicrobial properties as the positively charged –NH_3_ moieties impair the microbial cell membrane (leakage of intracellular constituents) by interacting with its negatively charged components [7]. However, the main weakness of Chi is its poor water solubility and inability to impede the formation of biofilm when deposited on the surface of an implant. In addition, the acidic conditions required for film formation are not compatible with physiological conditions. This flaw could limit the practical application of Chi-based coatings [8] but can be overcome by modifications or a combination with other agents. The incorporation of quaternary ammonium groups or grafting of polymer chains or small molecules onto the Chi backbone resulted in higher antimicrobial activity [9,10]. Adaptations also include treatment with polydopamine [10,11] and anionic polysaccharides such as heparin, hyaluronic acid (HA), alginate and pectin [10,12]. Moreover, from a medical perspective, it is extremely important to establish coatings with a drug release strategy from planar surfaces. Nanoparticle and microparticle polysaccharide-based polyelectrolyte complexes (PECs) are a promising alternative that can be prepared by simple complexation/coacervation of differently charged polysaccharides and have already been applied for the delivery of various therapeutic drugs with advantages, such as improved bioavailability, extending the in vivo half-life of a drug, protecting the drug from harsh conditions, and providing sustained or triggered release of the drug [13,14,15]. Notably, many PECs have been formulated with cationic Chi as mentioned to be the most interesting and usable polysaccharide and negatively charged polysaccharides, such as HA, chondroitin sulfate, heparin, alginate, carboxymethyl cellulose, or dextran sulfate [13]. For example, PEC nanoparticles loaded with vascular endothelial growth factors have been prepared using dextran sulfate and Chi [16]. 

However, there is a need for next-generation coatings to be multifunctional, integrating multiple bacterial control strategies, as well as releasing drugs or other agents locally and in a controlled manner into the biological environment, including biocidal effects, antifouling behavior, and biofilm inhibitory properties. This can be achieved by versatile polyelectrolyte complexes, where the structure and chemical character can be manipulated, from charge to structure, polarity to non-polarity, stability and thus drug delivery efficiency, etc. These properties can be modified by PECs, formed by combining biopolymers and surfactants [17], including various structures, for instance, micelles, gels, and precipitates. These structures are essential in cosmetics and pharmaceuticals as drug-delivery systems and especially as bioactive coatings [18,19,20,21]. Precipitation of PECs, formulated by polymer and oppositely charged surfactant is driven by electric charge and cooperative binding mechanism [21]. By varying the concentrations of the two components and thus the charge manipulation, different synergistic effects of the two substances involved can be obtained, leading to different physicochemical and structural properties of the complex [22,23]. These polyelectrolyte-surfactant complexes (PESCs) are of interest for many applications due to their characteristics to control rheology and to load/release active agents in a controlled manner. Using polyelectrolytes and surfactants, a wide range of delivery systems aimed at encapsulating bioactive compounds (e.g., association micelles, emulsions, biopolymer matrices) have been developed [24,25,26,27,28,29,30]. The main advantage attributed to surfactants in drug delivery is their function as absorption enhancers and enhancement of delivery of specific drugs in such a way that delayed release of drugs is achieved to prolong their duration of action [31].

Although there are several approaches for the development of smart coatings for medical devices, it is advantageous to establish a multifunctional PESCs based on the combination of surfactants with oppositely charged polyelectrolytes, providing multifunctionality, controlled release, and high specific surface area, resulting in surface properties with the desired bioactivity. This is still a relatively unexplored area and represents a major scientific challenge for material scientists working on coatings. 

A good example is successful research on the combination of HA and the cationic lysine-based surfactant *N*^ε^-myristoyl-lysine methyl ester (MKM), developed as PEC evaluating in vitro antimicrobial coatings for viscose (wound dressing) and urethral catheters [32,33]. This work prompts us to proceed with the preparation of such advanced biobased and bioactive PESC. To date, there have been no studies on the preparation and evaluation of a PESC using the cationic Chi (Chi) and the anionic lysine-based surfactant 77KS (Chi-77KS). This work investigates the potential multifunctional use of Chi-77KS as a drug delivery system by optional loading of amoxicillin (AMOX) in the micellar structures of PESC as a coating for polydimethylsiloxane (PDMS). Previous publications [34,35] show that the complex alone has promising biofilm inhibition properties as a coating for silicone materials, but there is still much to improve, especially towards biofouling properties. Therefore, here we investigated the properties of non-AMOX and AMOX-loaded coatings with an additional, second layer of coated HA on the model PDMS films. Among the various anionic polysaccharides, HA alone and in a composite form already has many potentials to lower the impact of biofilm-associated infections in diverse clinical settings due to its safety profile, biocompatibility, and anti-adhesive properties [36]. The physicochemical properties of these bilayer polysaccharides-based coatings attached to PDMS model films were investigated by X-ray photoelectron spectroscopy (XPS), water contact angle, and surface *ζ*-potential measurements. The biofilm inhibitory properties of such coated PDMS films were monitored using a quartz crystal microbalance with dissipation monitoring (QCM-D) by adsorption of a mixed-protein solution (Mix) of bovine serum albumin (BSA), fibrinogen (FIB), and γ-globulin (γG). 

Such architecture of formulations, combining drug embedding and antimicrobial as well as anti-biofilm properties represents a new generation of multifunctional bilayer architectural coatings that also exhibit good biofilm inhibiting and biocompatible properties for applicable silicone materials and thus can potentially be used as medical coatings.

## 2. Materials and Methods

### 2.1. Materials

The 77KS was provided by the Institute of Advanced Chemistry of Catalonia (Barcelona, Spain). Chi (50,000–190,000 Da, 75–85% deacetylated, product number 448869), AMOX (potency ≥ 900 μg/mg), glacial acetic acid (AcOH; ≥99.7%), toluene, phosphate-buffered saline (PBS; 10 mM phosphate buffer, 2.7 mM potassium chloride, and 137 mM sodium chloride), sodium hydroxide (5 M, pro analysis), BSA (lyophilized powder, ≥96%), FIB (90% clottable), γG (≥97%), PDMS (SYLGARD^®^ 184), L-glutamine (sterile-filtered, BioXtra), penicillin-streptomycin (0.1 μm filtered, BioReagent) and 3-(4,5-dimethylthiazol-2-yl)-2,5 diphenyltetrazolium bromide (MTT; BioReagent, ≥97.5% HPLC) were purchased by Sigma-Aldrich (Vienna, Austria). Hyaluronic acid sodium salt (low molecular weight 80,000–100,000 Da) was purchased from Carbosynth Limited (Compton, Berkshire, UK). Minimum Essential Medium and fetal bovine serum (FBS) were supplied from Fisher Scientific (Vienna, Asutria) and etoposide from Santa Cruz Biotechnology (Santa Cruz, CA, USA). Ultrapure water (resistivity of 18.2 MΩ cm at 25 °C) was prepared using the Milli-Q system (Millipore Corporation, Bedford, MA, USA). The sensors used for the QCM-D measurements were gold-coated quartz crystal sensors QSX301 purchased from Biolin Scientific (Gothenburg, Sweden). Gram-positive *Staphylococcus aureus* (*S. aureus*) ATCC 29213, Gram-negative *Escherichia coli* (*E. coli*) ATCC 25922, and the mouse fibroblast cell line (NCTC clone 929: CCL 1; L929) were obtained from the American Type Culture Collection (ATCC). Micro agar was supplied from Duchefa Biochemie (Haarlem, The Netherlands), Difco^TM^ tryptic soy agar (TSA) from Becton, Dickinson and Company (Le Pont de Claix, France), tryptic soy broth (TSB) from Biolife Italiana Srl (Milan, Italy) and ethanol (96% *V*/*V*, puriss.) from Honeywell (Seelze, Germany).

### 2.2. Preparation of Samples

In the first part, model PDMS films on QSX301 (Au-PDMS) were coated with two layers: a) Chi-77KS or Chi-77KS/AMOX and b) HA (Au-PDMS/Chi-77KS/HA and Au-PDMS/Chi-77KS/AMOX/HA). Model samples with their annotations are listed in Table 1. These samples were characterized for surface properties and antifouling affinity by protein adsorption capacity. In the second part, real samples on silicone discs were prepared and investigated for biofilm inhibitory properties and biocompatibility, as the most important parameters for the final application and medical usability. The annotations of real samples are listed in Table 2.

#### 2.2.1. Model PDMS Films: QCM-D Sensors and Modification

The thin PDMS films were spin-coated on quartz crystal sensors QSX301 (Au-PDMS) according to the procedure by Bračič et al. [37]. Briefly, a 10% (*w*/*w*) PDMS stock solution in toluene was prepared using a 10 (monomer): 1 (curing agent) ratio. For spin-coating, the PDMS stock solution was diluted with toluene to obtain a 0.5% (*w*/*w*) PDMS solution. Finally, 50 µL of 0.5% (*w*/*w*) PDMS solution was spin-coated onto QSX301 sensors (30 s at 4000 rpm, and 2500 rpm/s). Afterward, samples were kept in a vacuum oven at 70 °C for 2 h.

#### 2.2.2. Adsorption of Chi-77KS, Chi-77KS/AMOX and HA onto PDMS Films

The adsorption and stability of Chi-77KS, Chi-77KS/AMOX, on the Au-PDMS surfaces were explored by QCM-D (E4, Q-Sense, Biolin Scientific, Gothenburg, Sweden) at 25 ± 0.1 °C. The coated sensors were placed in the QCM-D flow cells, with subsequent equilibration in ultra-pure water to establish a stable baseline frequency (t ~ 120 min). In the next step, the experiments were restarted and a baseline in ultrapure water was set up. Subsequently, ultrapure water was exchanged with the formulation (Chi-77KS or Chi-77KS/AMOX; pH 6.5) or HA. A tubing pump (ISMATEC IPC 4, IDEX Corporation, Dietikon, Switzerland) with a flow rate of 0.1 mL/min was used throughout all experiments. The 3rd overtone was observed or both, resonance frequency (Δ*f*) and dissipation (Δ*D*). A decrease in Δ*f* signifies increased mass (adsorption), whereas higher Δ*D* indicates decreased rigidity of the coating. The details of this procedure are described in [34,35].

For the adsorption of HA on samples Au-PDMS/Chi-77KS and Au-PDMS/Chi-77KS/AMOX, these samples were mounted into the flow cells and equilibrated with ultra-pure water until stable resonance frequency was established. Following this, 0.2 wt% HA (in ultra-pure water; pH 7) was injected over the cells for 60 min, and subsequently rinsed with NaCl (150 mM) and with ultra-pure water (pH 7) for 30 min, respectively. The sample annotations are given in Table 1. 

#### 2.2.3. Real Samples: PDMS Discs

Real PDMS samples (discs) as representative medical material (*A* = 1.2 cm^2^, *h* = 1.5 mm) were made using a 10 (monomer):1(curing agent) ratio, and cured at 70 °C for 2 h. Formulations were coated with developed bilayer coatings (Table 2) by the dip-coating method drying under a stream of nitrogen. The list of PDMS sample abbreviations with descriptions can be found in Table 2.

### 2.3. Characterization Methods

#### 2.3.1. Surface Analyses

The wettability of PDMS films was characterized by water contact angle measurements (OCA 35 Optical Contact Angle Meter and SCA 20 version 4.1.12 software, DataPhysics Instruments, Filderstadt, Germany) using 3 μL drops of ultra-pure water at 25 °C in triplicates.

The elemental composition of solid samples was evaluated by XPS (PHI 5600, Physical Electronics, Inc., Chanhassen, MN, USA), equipped with an aluminum K_α_ X-ray source (h*ν* = 1486.6 eV) and a hemispherical electron analyzer under an ultra-high vacuum. XPS measurements were performed at a 45° take-off angle and an area with *r* = 0.4 mm. The C 1s peak, centered at 284.8 eV was used to correct the binding energy (*E*_B_) scale. The survey spectra were measured at 187.8 eV and the high-resolution spectra were measured at 29.35 eV with analyzer pass energies in constant analyzer energy mode. MultiPak 8.1c software was used for the spectra normalization, *E*_B_ correction, and background subtraction.

Streaming potential measurements were applied to determine the surface *ζ*-potential using an instrument SurPASS 3 and an adjustable gap cell with a circular cross-section for 14 mm discs (Anton Paar GmbH, Graz, Austria). A sample duplicate on the QCM-D sensor was fixed on the adjacent sample holders. The distance between samples was adjusted to 103 ± 4 µm during several rinse cycles with a dilute PBS. The pH dependence of the *ζ*-potential was determined in the range between the native pH 7.4 ± 0.2 of the dilute PBS solution and the isoelectric point (IEP) of the respective top layer of the PDMS thin-film coating. The results comprise of the dependence of the *ζ*-potential on the pH of aqueous solutions of different electrolytes, i.e., KCl with an ionic strength of 0.0024 mol/L and 0.01 mol/L, respectively, and dilute PBS. For all measurements, the pH of these aqueous solutions was adjusted starting at the native pH of the corresponding solution and terminating in the proximity of the IEP of the outermost layer of the thin-film coating. For individual samples and measurement conditions, the pH range was extended towards higher or lower pH. Measurements of the streaming potential were recorded in triplicate at each pH. Arbitrarily, the measurements were repeated for another set of samples to address the sample-to-sample reproducibility.

#### 2.3.2. Potentiometric Titrations

Potentiometric titration of proteins: HA, BSA, FIB and γG as macromolecular solutions was performed in triplicates as follows. The titration cell was filled with analyte solution of a known concentration and titrated (T70, Mettler Toledo, Greifensee, Switzerland) in the pH region 2.5–11.0 using 0.1 M HCl and 0.1 M KOH. Measurements were performed in an inert N_2_ atmosphere and at an ionic strength of 0.1 M KCl using the InLab Routine pH electrode (Mettler Toledo, Giessen, Germany). A detailed description of the method can be found elsewhere [38,39]. 

#### 2.3.3. Adsorption of Proteins

Samples Au-PDMS/Chi-77KS/HA and Au-PDMS/Chi-77KS/AMOX/HA were mounted in the QCM-D flow cell and initially equilibrated with ultra-pure water, and followed by PBS until establishing a stable baseline frequency. BSA, FIB and γG were dissolved in physiologically relevant PBS buffer (pH 7.4) at final *c* = 1 mg/mL. The Mix was injected over the samples for 60 min and subsequently rinsed with PBS solution for 60 min (*Q* = 0.1 mL/min). The protein adsorption study was performed at 37 ± 0.1 °C and pH 7.4 to mimic the physiological conditions. The adsorbed mass (*Γ*_QCM_), film thickness (*h*_f_), viscosity (*η*_f_), and elastic shear modulus (*μ*_f_) of the adsorbed layers were calculated using the Voigt model (QTools 3.0.12 software, Q-Sense, Biolin Scientific, Gothenburg, Sweden).

#### 2.3.4. Biofilm Formation Assay

The anti-biofilm action of coated real PDMS samples (discs) (PDMS/Chi-77KS, PDMS/Chi-77KS/AMOX) was evaluated comparatively to the non-coated PDMS discs. The Gram-negative bacterium *E. coli* and the Gram-positive bacterium *S. aureus* were used as prevailing and thus representative bacteria in biofilm formation. Biofilm formation assay was described in detail in our previous publication [35].

Briefly, neat and coated PDMS discs were incubated in 200 mL baffled Erlenmeyer flasks containing 50 mL TSB (37 °C and 180 rpm). Samples were removed from the flasks after 24 h and rinsed by sterile 1× PBS to detach the planktonic bacteria from the disc surface. In the next step, the individual discs were inserted into 15 mL centrifuge tubes containing 3 mL of TSB and vortexed for 10 min at 2000 rpm to disrupt and displace the biofilm from the samples. An aliquot of 100 µL from the obtained vortexed suspension of each sample was taken to prepare serial dilutions with 1× PBS (pH 7.4), spread on TSA in triplicates, and incubated at 37 °C. In this manner, after 24 h, bacteria that remained on the surfaces of the discs were assessed and expressed as colony-forming units per mL (CFU/mL).

#### 2.3.5. Biocompatibility Testing

Biocompatibility testing to determine biological reactivity and potential cytotoxic activity of the coatings Chi-77KS/HA and Chi-77KS/AMOX/HA on real PDMS samples was performed on mouse fibroblasts (L929 cells) in vitro. The cytotoxic activities of the PDMS coated disc extracts were determined after 24 ± 1 h of exposure with the MTT cytotoxicity assay according to the International Standards ISO 10993-5:2009, Biological evaluation of medical devices—Part 5: Tests for in vitro cytotoxicity [40]. In addition, the biological reactivity of the same cells following the exposure to the PDMS disc extracts for 24 ± 1 and 48 ± 1 h was observed visually by light microscopy and was graded on a scale of 0 to 4, according to ISO 10993-5:2009 [40].

Before the testing, the PDMS coated discs were sterilized by dipping them in 70% ethanol and drying them under sterile conditions. Extraction of the coated discs was conducted in 50 mL centrifuge tubes (Corning, Amsterdam, the Netherlands) under sterile conditions by incubating the PDMS coated discs in growth medium (Minimum Essential Medium, 1.25 cm^2^/mL) supplemented with 10% FBS, 4mM L-glutamine, 100 IU/mL penicillin and 100 µg/mL streptomycin, for 24 ± 1 h at 37 ± 1 °C and 5 ± 1% CO_2_ in a humidified atmosphere with shaking (100 rpm), as described in ISO 10993-12:2021 [41]. In parallel, the growth medium was incubated at 37 °C and 5 ± 1% CO_2_ in humidified atmosphere for 24 ± 1 h, to provide the same culturing conditions for the control group.

The L929 cells were seeded onto 96-well microplates (Nunc, Naperville, IL, USA) at a density of 4000 cells/well, which corresponds to 20,000 cells/mL, and were incubated for 24 ± 1 h at 37 ± 1 °C and 5 ± 1% CO_2_ in the humidified atmosphere to attach. On the next day, the cells were exposed to the extracts (100 *V*/*V*%) and dilutions (50, 25, 12.5 and 6.25 *V*/*V*% corresponding to 1.25, 0.625, 0.3125, 0.1563 and 0.0781 cm^2^/mL, respectively) for 24 and 48 ± 1 h at 37 ± 1 °C and 5 ± 1% CO_2_ in a humidified atmosphere. In the experiment, a vehicle control (growth media incubated under the same conditions that were used for the extraction of the PDMS coated discs; 24 ± 1 h hours at 37 ± 1 °C and 5 ± 1% CO_2_ in the humidified atmosphere), fresh complete growth media (negative control) and positive control (etoposide; 100 µg/mL) were included.

## 3. Results and Discussion

### 3.1. Influence of the Additional HA Layer on Antifouling Properties

Samples Au-PDMS/Chi-77KS and Au-PDMS/Chi-77KS/AMOX were characterized and these results have been published in detail previously [34,35]. Here, these films were additionally coated with a HA layer (as a second layer) to further improve the protein-repelling properties and approach zero protein adsorption, indicating antifouling efficiency. Below, the influence and effectiveness of the additionally adsorbed HA layer, along with properties of polysaccharide-based bilayer coatings for biofilm inhibiting surfaces are discussed.

#### 3.1.1. Adsorption and Characterization of the Additional HA Layer

**Figure 1:**
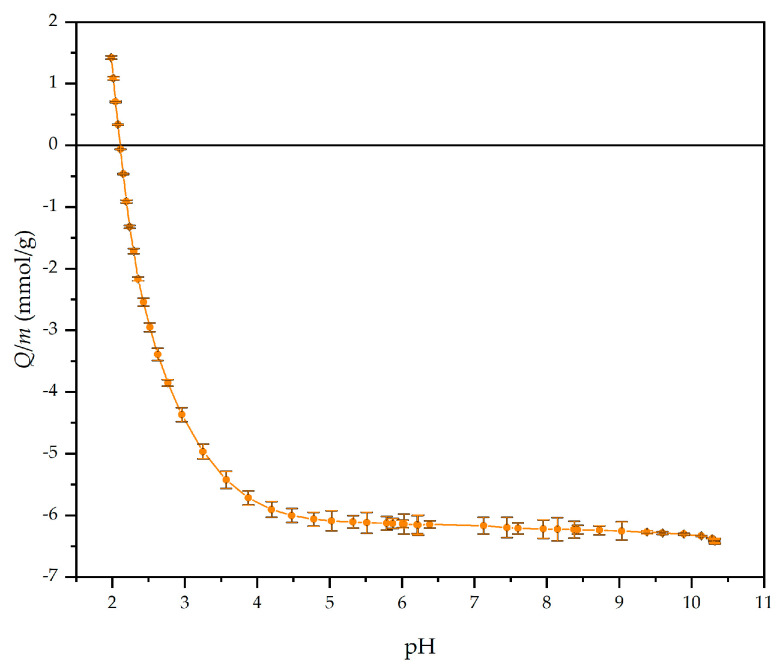
Potentiometric titration curve for HA with a charge per mass as a function of pH.

An anionic and highly hydrophilic biopolymer layer of HA can improve long-term stability, enhance antifouling properties comprehensively, and even diminish the growth of *Staphylococcus aureus* (*S. aureus*) [42]. As determined by pH-potentiometric titration (Figure 1), HA has a negative net charge at pH 7.0 with all of its carboxyl group’s deprotonated (pK_a_ ≈ 3.0–4.0 [43,44,45]). In this way, HA was adsorbed onto the Au-PDMS/Chi-77KS and Au-PDMS/Chi-77KS/AMOX surface at conditions in an extended chain conformation to ensure a homogeneous and thin adsorbed second layer. 

**Figure 2:**
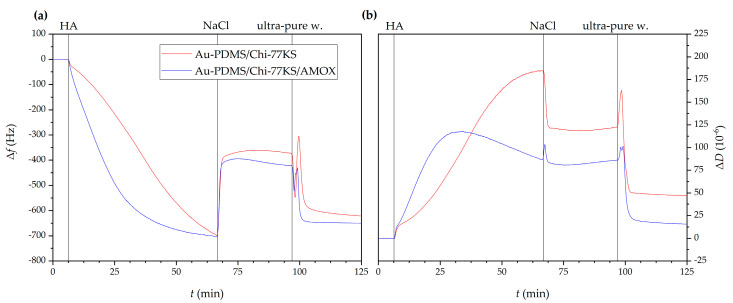
Changes in QCM-D frequency (**a**) and dissipation shift (**b**) as a function of time for the adsorption of HA onto Au-PDMS/Chi-77KS and Au-PDMS/Chi-77KS/AMOX surfaces.

Results of HA adsorption as the second layer monitored by QCM-D revealed similar behavior for both Au-PDMS/Chi-77KS and Au-PDMS/Chi-77KS/AMOX (Figure 2), as the frequency shift after 1 h for both samples decreased (Δ*f* = −700 Hz). After the rinsing step using 150 mM NaCl, frequency increased to Δ*f* = −370 Hz for Au-PDMS/Chi-77KS and to Δ*f* = −420 Hz for Au-PDMS/Chi-77KS/AMOX. The enormous change in frequency and dissipation might be explained by the fact that HA can absorb an immense amount of water and expand up to 1000-fold of its solid volume, forming a loose hydrated network [44].

Nevertheless, the increase in Δ*f* for both Au-PDMS/Chi-77KS/HA and Au-PDMS/Chi-77KS/AMOX/HA might also be explained by the effect of NaCl, altering density and packing HA molecules closer to the surface through charge screening. The latter was observed with Δ*D* decreasing from an initial 175 to 125 × 10^−6^ for Au-PDMS/Chi-77KS/HA and 120 to 85 × 10^−6^ for Au-PDMS/Chi-77KS/AMOX/HA after the NaCl rinsing step (Figure 2b). In the presence of NaCl, HA may adopt a coil-like structure on the surface, as repulsion forces between HA charges are reduced, leading to increased Δ*f* and decreased Δ*D* [42]. 

The subsequent and final rinsing step using ultra-pure water displayed a brief, burst-like decrease in Δ*f* and an increase in Δ*D*, followed by the same, but reversed behavior. This can be explained by a change in solution density (ultra-pure water is less dense than 150 mM NaCl) and/or surface water uptake, as explained before. Further rinsing of the surface with ultra-pure water probably leads to additional water uptake, or even HA taking different conformation, due to decreasing Δ*f* to a final −600 and −650 Hz for Au-PDMS/Chi-77KS/HA and Au-PDMS/Chi-77KS/AMOX/HA, respectively (Figure 2a). Despite the significant decrease in Δ*f* that indicated the increase of the mass on the surface, Δ*D* decreased additionally to final values of 50 × 10^−6^ for Au-PDMS/Chi-77KS and 10 × 10^−6^ for Au-PDMS/Chi-77KS/AMOX (Figure 2b) Such behavior revealed that the surface might be subjected to structural changes, resulting in a less viscous layer. The desorption results indicated the stability of the adsorbed HA layer onto films previously coated with the complex Chi-77KS and the complex with embedded drug, Chi-77KS/AMOX, and suggested possible physical interactions between adsorbate and adsorbent (Van der Waals forces including hydrogen bonds). 

**Figure 3:**
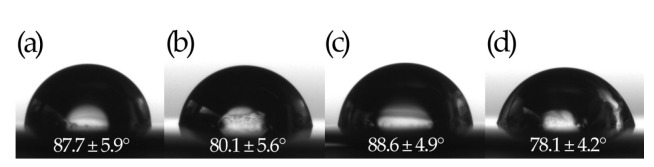
Water contact angles of (**a**) Au-PDMS/Chi-77KS [35], (**b**) Au-PDMS/Chi-77KS/HA, (**c**) Au-PDMS/Chi-77KS/AMOX [35] and (**d**) Au-PDMS/Chi-77KS/HA/AMOX/HA.

HA-coated surfaces are more hydrophilic with Au-PDMS/Chi-77KS/HA (80.1 ± 5.6)° having a lower water contact angle compared to Au-PDMS/Chi-77KS (87.7 ± 5.9)° [35], and Au-PDMS/Chi-77KS/AMOX/HA (78.1 ± 4.2)° having a lower water contact angle compared to Au-PDMS/Chi-77KS/AMOX (88.6 ± 4.9)° [35] (Figure 3). The reference PDMS film shows a hydrophobic character and a contact angle of (111.4 ± 0.6)°.

The deposition of an additional HA layer introduced an even more hydrophilic character of the bilayer coating, which was expected due to the considerable wetting properties of HA. Better wettability is essential for lower friction in medical devices, and HA shows a prominent application as hydrophilic coatings for a plethora of medical devices, e.g., catheters, guidewires, and sensors. Moreover, HA-based and hydrophilic coatings on medical devices can significantly minimize fouling, improve biocompatibility and lubricity (lower tissue abrasion), which are essential characteristics when considering usability and advances of the healing process [46].

**Figure 4:**
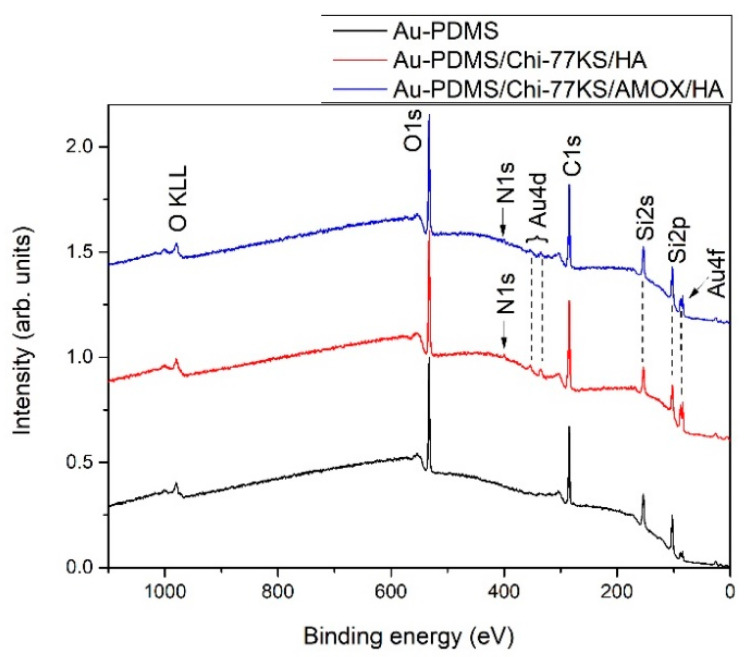
Survey XPS spectra of Au-PDMS, Au-PDMS/Chi-77KS/HA, and Au-PDMS/Chi-77KS/AMOX/HA.

The survey XPS spectra for samples Au-PDMS/Chi-77KS/HA and Au-PDMS/Chi-77KS/AMOX/HA are shown in Figure 4 to further estimate the adsorption of HA as a second layer. As a reference, also the spectrum for Au-PDMS is shown, whilst the elemental composition of Au-PDMS/Chi-77KS and Au-PDMS/Chi-77KS/AMOX was discussed previously [34]. The spectra of both samples with the coatings have distinctive five photoelectron peaks, corresponding to electrons originating from C, O, N, Si and Au. The intensity of Au 4d and Au 4f peaks is very small and they originate from QCM-D sensors, whereas Si 2s and Au 2p peaks are resulting from PDMS coating. Since all coatings (PDMS as well as Chi, 77KS, AMOX and HA) contribute to the presence of C 1s and O 1s peaks, the evidence of the presence of the upper organic coatings is the presence of N 1s, originating from Chi, 77KS and AMOX. Because the at.% of N in organic formulations in contrast to other elements is relatively low, the intensity of N 1s peak is very low. The concentration of nitrogen in surface films is thus only approximately 2 at.%. This effect is even more pronounced in the case of sulfur that we would expect to see because of the presence of AMOX, however, it cannot be observed because its concentration is lower than the detection limit of XPS. However, it has been shown previously with time-of-flight secondary ion mass spectrometry (ToF-SIMS) that AMOX is available on the coated PDMS surfaces [34]. From Figure 4 we can also conclude that the deposited layers are very thin since Au from the substrate is visible in the spectra, suggesting that the applied coating is probably thinner than 5 nm (it should be noted that the analyzed depth for XPS analyses is about 5 nm).

**Figure 5:**
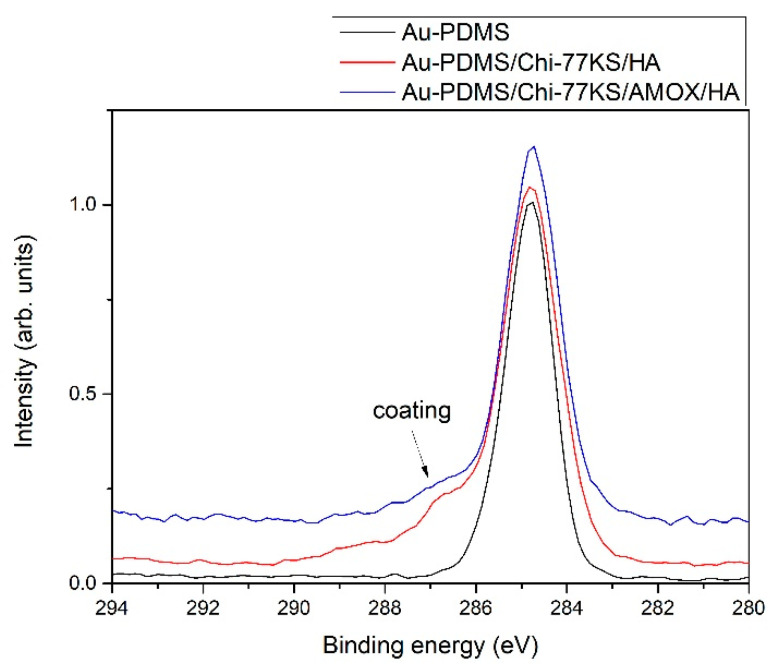
Comparison of high-resolution XPS spectra of carbon C1s of Au-PDMS, Au-PDMS/Chi-77KS/HA, and Au-PDMS/Chi-77KS/AMOX/HA.

To evaluate the presence of the coatings on PDMS, we show in Figure 5 a comparison of high-resolution carbon spectra of PDMS before and after applying the coatings. The carbon spectrum for PDMS shows only one symmetrical peak, which is typical for PDMS. After applying the coatings, a noticeable shoulder appears on the high-binding energy side of the carbon spectrum (marked with an arrow), which is a consequence of the presence of the coatings that are rich with various functional groups.

**Figure 6:**
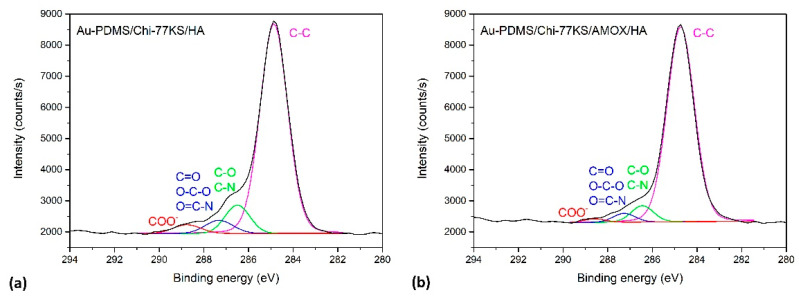
High-resolution XPS deconvoluted spectra of (**a**) Au-PDMS/Chi-77KS/HA and (**b**) Au-PDMS/Chi-77KS/AMOX/HA.

The detailed high-resolution spectra of the coatings are further shown in Figure 6 for Au-PDMS/Chi-77KS/HA and Au-PDMS/Chi-77KS/AMOX/HA. We should note, that organic substances such as Chi, 77KS, AMOX and HA contain several different groups that can be present in all of the mentioned organic formulations. Therefore, we cannot explicitly distinguish the contributions of different substances. As shown before in Figure 5 for Au-PDMS, a symmetrical C 1s peak at a binding energy ~284.8 eV is observed, corresponding to the –CH_3_ moieties in PDMS and other C–C, C–H and C–Si-containing species [47,48]. However, Figure 6 clearly shows, that after applying the coatings, the presence of Chi, 77KS, AMOX and HA (Au-PDMS/Chi-77KS/HA and Au-PDMS/Chi-77KS/AMOX/HA) produces a feature due to COO^−^ species, several features on the high binding energy side of the main peak due to the C–O/C–N-containing species, and to a lower extent also a feature at the position characteristic for O–C–O/C=O/C–N=O species [34]. The latter is an indication of HA adsorption that carries carboxyl groups. Here, we should also note that the shoulder on the high-binding energy side is more pronounced for the sample Au-PDMS/Chi-77KS/HA shown in Figure 6a than for the sample Au-PDMS/Chi-77KS/AMOX/HA shown in Figure 5 and Figure 6. This is due to the presence of AMOX in the sample Au-PDMS/Chi-77KS/AMOX/HA (Figure 6b), which contains much fewer C–O groups than HA. Thus, the C–O peak is less pronounced.

**Figure 7:**
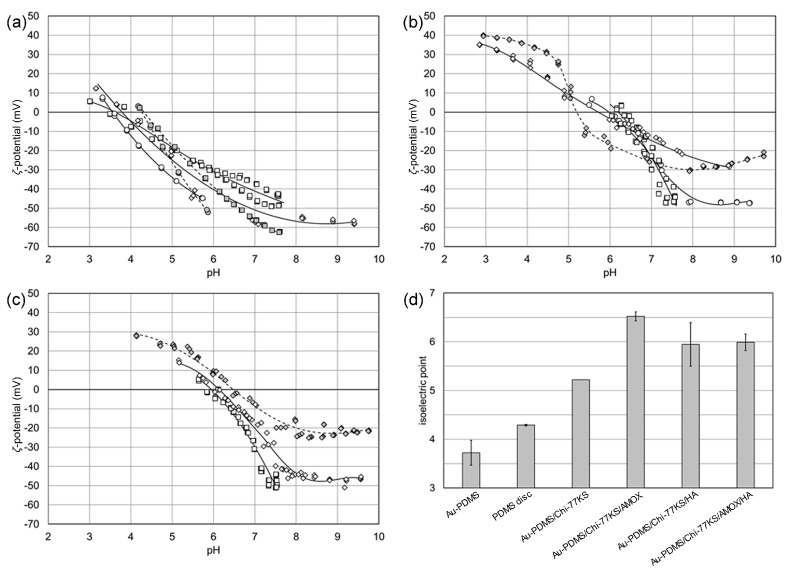
pH dependence of *ζ*-potential for (**a**) PDMS thin-film coating on gold sensors (empty symbols) and PDMS disc (filled symbols), (**b**) Au-PDMS/Chi-77KS (filled symbols) and Au-PDMS/Chi-77KS/HA (empty symbols), (**c**) Au-PDMS/Chi-77KS/AMOX (filled symbols) and Au-PDMS/Chi-77KS/AMOX/HA (empty symbols), and (**d**) isoelectric points. Square symbols represent the measurement in PBS, circles in 0.0024 mol/L KCl, and diamonds in 0.01 mol/L KCl.

Figure 7 shows the results of the zeta (*ζ*)-potential analyses of thin-film coatings on QCM-D gold sensors with different terminal layers, i.e., PDMS, Chi-77KS, AMOX, and HA. All measurements were performed using PBS and KCl. Results obtained for samples with additional HA layer (Au-PDMS/Chi-77KS/HA and Au-PDMS/Chi-77KS/AMOX/HA) and casted PDMS discs using both PBS and KCl were compared to results obtained for samples Au-PDMS, Au-PDMS/Chi-77KS and Au-PDMS/Chi-77KS/AMOX using only KCl from the previous study [35]. The pH dependence of the *ζ*-potential for the PDMS thin-film coating on a gold sensor in Figure 7a is compared with the results obtained for a casted PDMS disc. The different electrolyte solutions used appear responsible for the deviation in the *ζ*-potential above the IEP at pH 3.7 ± 0.25 for the PDMS thin-film coating and at pH 4.3 ± 0.02 for the PDMS disc. The increase in *ζ*-potential (decrease in the magnitude of the *ζ*-potential) is expected when raising the ionic strength of KCl from 0.0024 mol/L to 0.01 mol/L. According to the model of the electric double layer, the decrease in the magnitude of the *ζ*-potential is explained by a compression of the diffuse layer represented by the Debye length [49]. The deviation in the *ζ*-potential determined in 0.0024 mol/L KCl and dilute PBS (with the conductivity adjusted to match a 0.0024 mol/L KCl solution) is not as obvious. Although it may be expected that the divalent HPO_4_^2−^ ion may selectively adsorb at the PDMS-water interface thereby driving this interface more acidic, the missing effect on the IEP excludes the assumption of specific ion adsorption. The higher ionic strength of the dilute PBS solution of 0.004 mol/L compared to 0.0024 mol/L for the aqueous KCl solution superimposes the alike electric conductivity. What remains unclear is the lower magnitude of the *ζ*-potential determined in dilute PBS compared to 0.01 mol/L KCl.

The subsequent layers of either Chi-77KS or Chi-77KS covered with HA behave more complex. For Au-PDMS/Chi-77KS we find a distinct point of inflection in the pH dependence of the *ζ*-potential, which coincides with the IEP at pH 5.2. The expected IEP for Chi with a degree of acetylation of 75–80% is located in the range of pH 8–9 [50]. The formation of a complex between Chi and the lysine-based surfactant 77KS is responsible for the shift of the IEP and the transfer of a basic to an amphoteric behavior of the thin-film coating. The carboxylic acid group of 77KS likely combines with the protonated amine groups of Chi, thereby forming a stable complex. On the other hand, this mechanism of complex formation consumes available basic NH_2_ groups of Chi, which finally weakens the basicity of the polysaccharide–surfactant complex.

When applying the coating of HA, the IEP shifts to slightly higher pH 5.9 ± 0.45, which contradicts the expectation derived from the acidic behavior of HA and the result of the potentiometric titration with a point-of-zero-charge at pH 2. Even if we assume a further compensation of the remaining basic NH_2_ entities of Chi by the carboxylic acid groups of HA, we expect a shift of the IEP to lower pH.

Another explanation for this unexpected shift in the IEP considers an instability of the thin-film coating occurring during the long-time exposure to the aqueous KCl solution (the recording of the *ζ*-potential by the streaming potential method requires equilibration of the material surface for at least 10 min). The extremely reproducible measurements of various pairs of Chi-77KS/HA-coated sensor disks in PBS, as well as in KCl with an ionic strength of 0.0024 mol/L and 0.01 mol/L, respectively, contradict the assumption of dissolution or disintegration of the thin-film coating. Furthermore, the deposition of HA on the Chi-77KS/AMOX-terminating thin-film coating gives the same IEP at pH 6.0 ± 0.17. Note that the IEPs for HA-terminating coatings of either Chi-77KS or Chi-77KS/AMOX are approached from opposite ranges of pH.

The effect of AMOX on the *ζ*-potential and IEP of Chi for Chi-77KS introduces a similar challenge in the interpretation of the observed results as the terminal HA coating does. AMOX contains a carboxylic acid group and a primary amine group, which are available for dissociation and protonation, respectively, and for an electrostatically driven binding to the primary amine groups of Chi. The carboxylic acid group is consumed by binding to the amine groups of Chi whereas the remaining amine groups of AMOX are available for the interaction with water, thereby introducing a positive charge. As mentioned above, the coating with HA partially compensates the positive charge leading to the same net amphoteric surface as for Chi-77KS/HA.

To better understand the influence of the terminating HA layer on the *ζ*-potential of the corresponding coatings, we compare the results shown in Figure 7b,c with the *ζ*-potential obtained for similar polysaccharide films that are reported in the literature. Richert et al. observed an alternating *ζ*-potential of a Chi-HA multilayer with *ζ* = + 50 … + 70 mV (at pH 5) for Chi-terminated coatings and *ζ* = − 35 … − 20 mV for coatings terminated by hyaluronic acid [12]. Although they used a buffer solution with a significantly lower ionic strength, giving rise to a higher magnitude of the *ζ*-potential, the apparent charge reversal is missing when comparing their results with the *ζ*-potential of the coatings terminated by Chi-77KS (*ζ* = +10 mV, pH 5) and Chi-77KS/HA (*ζ* = +15 mV) in Figure 7b. On the other hand, Sandri et al. reported IEPs at pH < 4 for Chi and Chi/HA embedded in a matrix of pullulan [51]. It is likely that the embedment of the cationic and anionic polysaccharides in the pullulan matrix prevents exposure of functional groups to water and thus the formation of additional surface charge. Zemljič et al. reported the *ζ*-potential and IEPs of coatings made of complexes of Chi and polyphenols [52]. Depending on the support (polyethylene or polypropylene films), they found either negligible or significant shifts of the IEPs for Chi and Chi-polyphenol complexes. We conclude a similar behavior of the coatings Chi-77KS and Chi-77KS/AMOX. The diminishing effect of the additional terminal coating by HA on the *ζ*-potential suggests a complexation reaction and an embedment of HA in the already existing Chi-77KS and Chi-KS/AMOX layers. 

This assumption is supported by the changes in the water contact angle. Like the analysis of the *ζ*-potential, the water contact angle measurement focuses on the properties of the interface between the surface coating and water and thus on the outermost solid surface. The changes from 88° for either Chi-77KS or Chi-77KS/AMOX to 78–80° after coating with HA indicate a marginal increase in surface hydrophilicity. However, these contact angles are much larger than those observed for pure coatings of HA [53]. 

The mentioned complexation of HA with the first attached layer significantly reduces the number of polar groups due to hydrogen bonding and electrostatic interactions, and the key to minor differences still proved that some of the introduced HA polar groups contribute to the surfaces, as also shown by other techniques and coating efficiency.

#### 3.1.2. Adsorption of Mixed-Protein Solution

**Figure 8:**
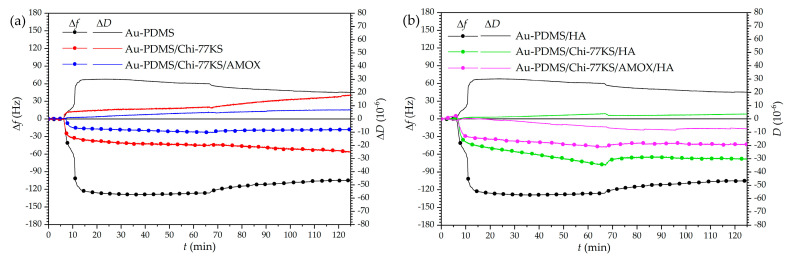
Changes in QCM-D frequency (**a**) and dissipation shift (**b**) as a function of time for the adsorption of the Mix onto Au-PDMS, Au-PDMS/Chi-77KS, Au-PDMS/Chi-77KS/AMOX [35], Au-PDMS/Chi-77KS/HA, and Au-PDMS/Chi-77KS/AMOX/HA surfaces.

Competitive adsorption was studied among BSA, FIB and γG on different surfaces from Mix to mimic in vivo protein adsorption. Competitive adsorption of proteins is highly influenced by interprotein relations, their concentration, affinity towards the definite surface, and post-adsorption conformation [54]. BSA, FIB and γG in Mix do not agglomerate and sustain stable, as well as clear, solution throughout all experiments. The results obtained were evaluated and compared with the previous study [35] in Figure 8 and Figure 10, and Table 3 and Table 4.

Adsorption profiles of Mix on Au-PDMS/Chi-77KS/HA and Au-PDMS/Chi-77KS/AMOX/HA coatings (Figure 8) showed that the first adsorption stage was quite rapid, suggesting that there was a larger attractive force between the surface and protein molecules. 

Although the layer of HA improved protein-repelling behavior for Au-PDMS/Chi-77KS/HA (Δ*f* = −29.9 Hz), no considerable difference was observed for Au-PDMS/Chi-77KS/AMOX/HA, as the behavior and the Δ*f* remained similar (Δ*f* = −19.1 Hz) as compared to Au-PDMS/Chi-77KS/AMOX with Δ*f* = −18.1 Hz (Figure 8a). The Δ*f* difference between Au-PDMS/Chi-77KS and Au-PDMS/Chi-77KS/HA was 26.5 Hz, which improved protein-repellent behavior significantly compared to Au-PDMS/Chi-77KS. 

**Figure 9:**
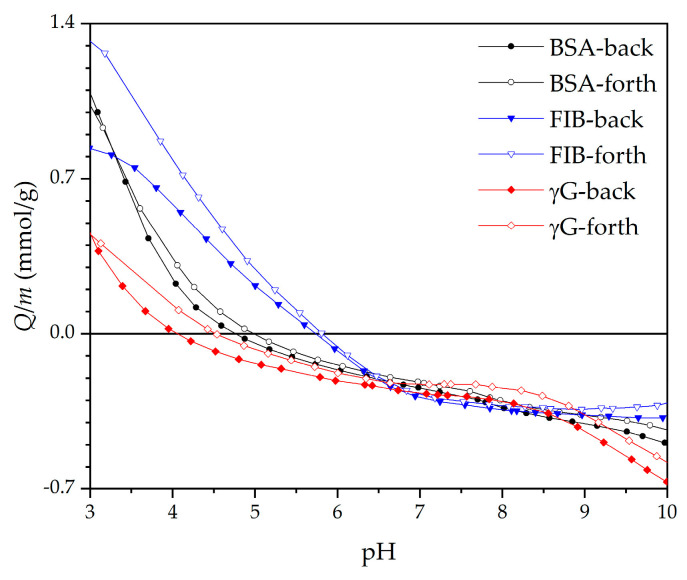
Potentiometric titration of BSA, FIB and γG [35].

Improved antifouling proteins properties were expected somehow whilst on these films with additional attached HA layer more anionic characters should be presented. BSA, FIB and γG exhibit an anionic charge at adsorption conditions (pH = 7, Figure 9) and thus electrostatic repulsion may occur [35]. Because after adsorption of HA only a negligible amount of carboxylic acids is free and available as anionic charge (low anionic charge density and much higher IEP as expected, see Figure 7), physical interaction may occur among proteins and coatings. According to DLVO theory, summarized repulsion and attraction potential define the total potential energy. Van der Waals forces contribute to interactions and thus the present physical interactions allow proteins to bind onto the surface and may dominate over repulsion forces.

Thus, adsorption of the Mix onto Au-PDMS/Chi-77KS/AMOX and Au-PDMS/Chi-77KS/HA showed fast adsorption at the beginning, followed by diminished Δ*f*. The surface became more rigid, as Δ*D* lowered (Table 3), although Δ*f* indicates notable desorption. It is more probable, that the change in dissipation results from higher viscosity/density of Mix, compared to single-protein solutions. The surface gradually continued to turn softer due to different protein conformations on the surface.

The Mix behaved like individual BSA molecules (as discussed in a previous publication [35]) when adsorbing onto Au-PDMS/Chi-77KS/AMOX/HA, with binding to the surface physically with the simultaneous expelling of water from the hydrophilic surface. 

It was realized that in this mixture, BSA may play the leading role within the Mix, as the adsorption trend indicated that the molecules were packing to the surface and making it more rigid, in the familiar fashion of single BSA molecules [35]. Additionally, the negative Δ*D* trend continued, even after the rinsing step, as more hydrophobic molecules packed even tighter to the surface to avoid the water. 

The Δ*D*/Δ*f* ratio (Table 3) shows that the adsorbed mixed-protein layer on Au-PDMS/Chi-77KS/AMOX (3.9 × 10^−7^ Hz^−1^) and Au-PDMS/Chi-77KS/AMOX/HA (3.9 × 10^−7^ Hz^−1^) is, similarly, viscous/hydrated. On the other hand, Au-PDMS/Chi-77KS/HA is less viscous or hydrated (1.2 × 10^−7^ Hz^−1^) than the non-coated Au-PDMS/Chi-77KS (3.2 × 10^−7^ Hz^−1^). The estimated wet layer thickness of mixed-proteins adsorbed on Au-PDMS/Chi-77KS/HA and Au-PDMS/Chi-77KS/AMOX/HA (Table 4) is significantly lower, with 8.00 ± 0.54 nm and 3.42 ± 0.12 nm, respectively, in comparison to Au-PDMS/Chi-77KS and Au-PDMS/Chi-77KS/AMOX with 25.50 ± 1.62 nm and 23.08 ± 2.08 nm, respectively. This is due to different conformations as a result of the interaction of the different surfaces with proteins.

**Figure 10:**
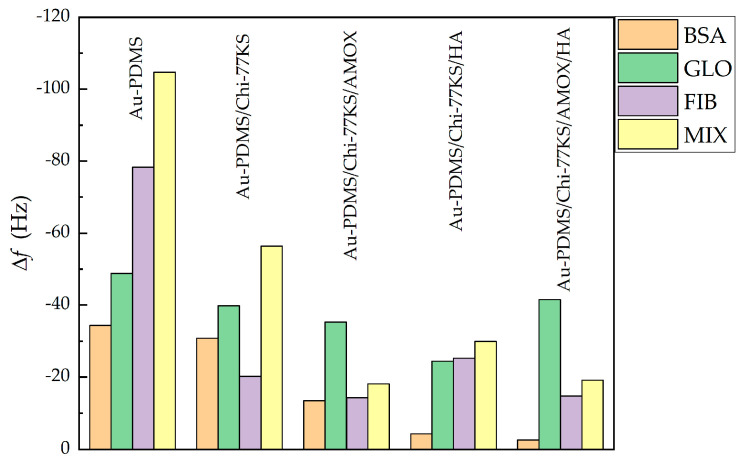
Δ*f* of BSA, FIB, γG and Mix after the rinsing step for Au-PDMS, Au-PDMS/Chi-77KS, Au-PDMS/AMOX [35], Au-PDMS/Chi-77KS/HA and Au-PDMS/Chi-77KS/AMOX/HA.

The comparison of single and mixed-protein adsorption behavior regarding the additional HA layer is shown in Figure 10. The additional HA layer on Au-PDMS/Chi-77KS/HA and Au-PDMS/Chi-77KS/AMOX/HA improved BSA-repellent behavior significantly. The adsorption of FIB on Au-PDMS/Chi-77KS/HA was slightly higher (Δ*f* = −25.2 Hz) when compared to Au-PDMS/Chi-77KS (Δ*f* = −20.2 Hz), while the FIB-repellence of the Au-PDMS/Chi-77KS/AMOX (Δ*f* = −14.3 Hz) stayed similar as for the Au-PDMS/Chi-77KS/AMOX/HA (Δ*f* = −14.8 Hz). 

The same behavior was observed for the adsorption of the Mix with Δ*f* of −18.8 Hz for Au-PDMS/Chi-77KS/AMOX and −19.1 Hz for Au-PDMS/Chi-77KS/AMOX/HA, as the Δ*f* for both remained similar after the rinsing step. In comparison, the HA layer on Au-PDMS/Chi-77KS/AMOX (Au-PDMS/Chi-77KS/AMOX/HA) did not improve FIB and Mix repelling properties significantly. On the other hand, the HA layer on Au-PDMS/Chi-77KS/HA improved γG-repellent behavior (Δ*f* = −24.4 Hz) in comparison to Au-PDMS/Chi-77KS (Δ*f* = −39.8 Hz). 

To be able to understand the protein adsorption, which occurs on the surface interface, the impact of surface parameters on the adsorption of proteins was analyzed. Generally, Δ*f* follows water contact angle and surface *ζ*-potential. A smaller water contact angle, more positive *ζ*-potential led to relatively more protein-repellent behavior. The affinity of proteins to adsorb to the surfaces from highest to lowest was as follows: BSA < FIB < γG < Mix. Importantly, adsorption of Mix lowered in all cases, especially when the additional HA layer was applied (Au-PDMS/Chi-77KS/HA and Au-PDMS/Chi-77KS/AMOX/HA). Adsorption of proteins and bacteria in the human body is a complex process, including various molecules (especially proteins) adsorbing and competing for the surface simultaneously. Thus, the demonstrated ability of functionalized surfaces to repel Mix is of paramount importance, and proof that coatings have the potential to be effective in the complex and demanding physiological environments in the human body. Due to this fact, the real coatings were carried out and tested by in vitro microbiological test to get an opinion about their potential real applications. 

### 3.2. Real Samples

The designation real sample stands for the sample, were made fully out of the medical-grade material, instead of such biomaterial being coated on a specific carrier (for example QCM-D sensor) to characterize its properties. The use of the real samples allows mimicking selected medical devices to explore their applicability in more realistic settings. Considering the common use of PDMS in various medical devices, it was chosen for evaluation in the form of discs. Due to promising results with the model samples, the real samples were assessed to demonstrate the applicability of novel coatings not only on the model but as well on the real surfaces. For this purpose, anti-biofilm properties and biocompatibility were evaluated to obtain data on potential medical usability. These two properties are essential for practical use in medicine. 

#### 3.2.1. Anti-Biofilm Capacity of Coated Discs

Blank (PDMS) and coated (PDMS/Chi-77KS, PDMS/Chi-77KS/AMOX, PDMS/Chi-77KS/HA and PDMS/Chi-77KS/AMOX/HA) discs were used for the microbiological study of biofilm-forming bacteria attached to the surface.

**Figure 11:**
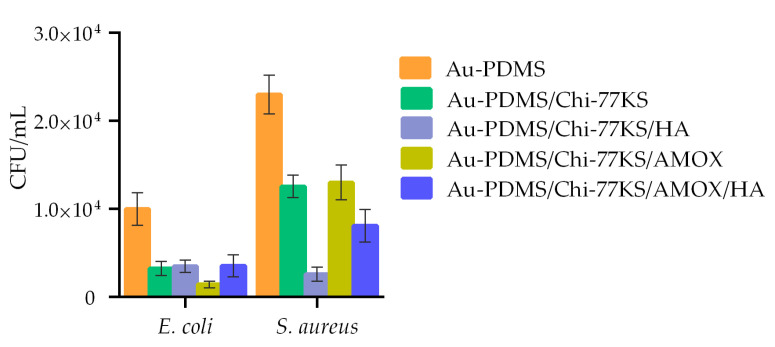
Attachment of *E. coli* and *S. aureus* and early biofilm formation on samples Au-PDMS, Au-PDMS/Chi-77KS and Au-PDMS/Chi-77KS/AMOX [35], and samples with an additional HA layer (Au-PDMS/Chi-77KS/HA and Au-PDMS/Chi-77KS/AMOX/HA) after 24 h of incubation.

The concentration of viable bacteria (*E. coli* and *S. aureus*) in CFU/mL, present on the PDMS surface was evaluated after 24 h of disc incubation with each bacterium. The difference in the bacteria count on the coated sample surface compared to the non-coated was significantly different. Results with an additional HA layer were compared with the previous study [35]. All coatings showed anti-biofilm action (Figure 11) with *S. aureus* having a higher affinity (colony count) to attach to the surface in comparison to *E. coli*. 

The number of *S. aureus* cells attached to PDMS/Chi-77KS was 45% and of *E. coli* 62% lower in comparison to the non-coated surface, demonstrating the considerable anti-biofilm action of the Chi-77KS coating. Particularly, PDMS/Chi-77KS/AMOX exhibited the most significant decrease in *E. coli* attachment and growth (81% reduced colony count) but diminished the growth of *S. aureus* the least (43% reduced colony count) considering the non-coated PDMS.

The additional HA layer on PDMS/Chi-77KS/HA improved *S. aureus* repelling behavior significantly by 40%, with a total of 85% lower colony-forming units in comparison to PDMS, with just a slight improvement of 2% against *E. coli* with 64% less attached bacteria. Sample PDMS/Chi-77KS/AMOX/HA obstructed attachment of *S. aureus* at 65% and *E. coli* at 75% compared to the blank PDMS surface. Considering sample PDMS/Chi-77KS/AMOX, these results show improvement in the anti-biofilm action against *S. aureus* (22% lower colony count). Both coatings with additional HA layer (PDMS/Chi-77KS/HA and PDMS/Chi-77KS/AMOX/HA) significantly diminished the attachment of bacteria and formation of biofilm in real systems in comparison to the non-coated PDMS samples.

Importantly, besides the introduction of an anti-biofilm character, these bilayer coatings improved hydrophilicity, which is one of the most important characteristics of designed coatings on medical devices. Lower contact angle improves lubricious quality and allows for blood that comes into contact with tubing and devices protected by these coatings to just flow (or wick) right over and around them without any impediments. These coatings reduce friction, allowing for ease of insertion while ensuring that they will not cause blood clotting around the device and will remain in the body for the necessary length of time [55]. 

#### 3.2.2. Biocompatibility Assays

**Figure 12:**
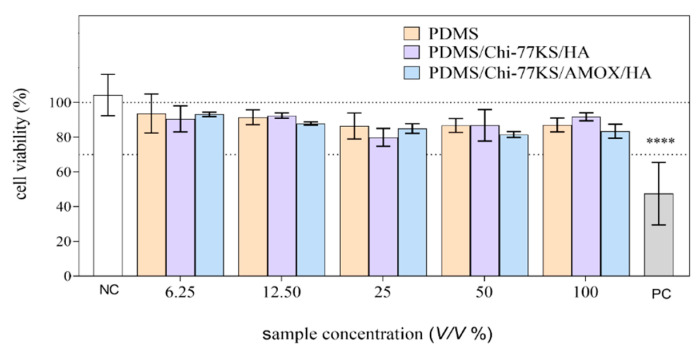
Viability of L929 cells after 24 ± 1 h of exposure to the PDMS disc extracts (6.25, 12.5 25, 50 and 100 *V*/*V*% corresponding to 0.0781, 0.1563, 0.3125, 0.625 and 1.25 cm^2^/mL, respectively). Data are presented relative to the vehicle control (VC)–growth medium, incubated under extraction conditions. Fresh growth media was used as the negative control (NC) and etoposide (100 µg/mL) as the positive control (PC). The asterisks denote a statistically significant difference between the sample and VC (**** *p* < 0.001, ANOVA).

Cytotoxicity evaluation of the PDMS samples was performed with the MTT assay after 24 ± 1 h of exposure of L929 cells to the PDMS disc extracts (6.25, 12.5, 25, 50 and 100 *V*/*V*%) according to the ISO 10993-5:2009 [40] (Figure 12). 

At the highest tested concentration of the extract, 100% (*V*/*V*) the cell viability was reduced for 13.0 ± 4.0, 8.3 ± 7.5, and 16.5 ± 4.1 on average after 24 h of exposure to PDMS, PDMS/Chi-77KS/HA and PDMS/Chi-77KS/AMOX/HA, respectively. However, the reduction was not statistically significant (ANOVA). According to the International Standard ISO 10993-5:2009 [40], the sample is considered cytotoxic when more than a 30% reduction of viability is determined after 24 ± 1 h of exposure. 

**Figure 13:**
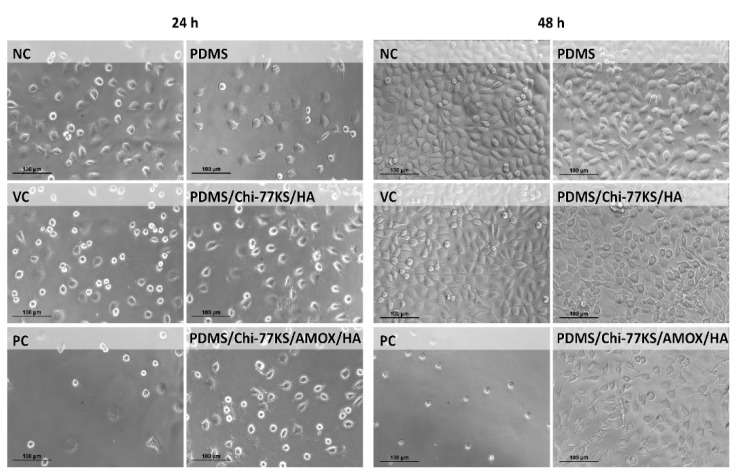
Morphology of L929 cells after 24 ± 1 and 48 ± 1 h of exposure to the PDMS disc extracts (100 *V*/*V*% corresponding to 1.25 cm^2^/mL, respectively). NC—negative control, fresh growth media; VC—growth medium, incubated under extraction conditions; PC—positive control, etoposide (100 µg/mL). Micrographs were taken under a light microscope (phase contrast) at 10 × 20 times magnification.

The biological reactivity of the PDMS samples was evaluated under the microscope after 24 ± 1 and 48 ± 1 h of exposure, according to the ISO 10993-5:2009 [40]. None or only slight or mild morphological changes (Grade 0–2) of the cells (L929) exposed to the PDMS disc extracts could be observed (Table 5, Figure 13).

There were no observable morphological changes between the VC cell population and the cells exposed to the PDMS and PDMS/Chi-77KS/HA extracts (Grade 0–1). A slightly higher grade (1–2) was observed after exposure to the two highest concentrations of the PDMS/Chi-77KS/AMOX/HA extract (50 and 100% (*V*/*V*)). This grade was assigned due to a lower density compared to the control cell population. However, based on the criteria of the ISO 10993-5:2009 guideline [40], the PDMS samples met the requirements of the biological reactivity test (biological reactivity was not greater than Grade 2).

Based on our results and the criteria of ISO 10993-5:2009 [40], it can be concluded that the coatings Chi-77KS/HA and Chi-77KS/AMOX/HA were not cytotoxic for L929 cells under the conditions applied and may be used for medical devices.

## 4. Conclusions

An additional coating of hyaluronic acid (HA) has been shown to improve the properties of protein-repellent and anti-biofilm surfaces, based on a highly hydrated negatively charged hydrophilic layer resulting from a combination of steric repulsive interactions and surface hydration. Due to the unique properties of HA, a layer of HA was additionally applied to the Au-PDMS/Chi-77KS/HA and Au-PDMS/Chi-77KS/AMOX/HA samples, to further improve the protein-repelling behavior. The additional HA layer was confirmed by X-ray photoelectron spectroscopy (XPS) and surface zeta (*ζ*)-potential measurements. It was observed that both samples acquired more hydrophilic character, with water contact angle changing from 87.7 ± 5.9° to 80.1 ± 5.6° for Au-PDMS/Chi-77KS/HA, and from 88.6 ± 4.9° to 78.1 ± 4.2° for Au-PDMS/Chi-77KS/AMOX/HA.

While the additional HA layer significantly improved the repulsion of Mix from the Au-PDMS/Chi-77KS/HA sample, that was not the case for the sample Au-PDMS/Chi-77KS/AMOX/HA. Therefore, AMOX is dominating over HA. The general observation in protein adsorption followed the trend that a more hydrophilic surface and an increase in the surface *ζ*-potential lead to improved protein-repellence. In this particular case, a single parameter does not strongly affect the biofilm attachment, which is apparently not linearly affected by wettability and is affected by other parameters, such as *ζ*-potential as well. Good protein-repellent properties might originate from the hydrated layer, the combination of surface hydrophilicity, and relatively homogenous distribution of charges on the surface.

The paper also deals with real samples simulating the material surface of medical devices, namely PDMS (as discs). A microbiological study was performed to determine the anti-biofilm action on real PDMS samples. The coatings reduced the incidence of biofilm formation in all samples significantly, with further improvement in the case of an additional layer of HA (except for Gram-negative *E. coli*). Moreover, coatings Chi-77KS/HA and Chi-77KS/AMOX/HA were biocompatible (non-cytotoxic), based on the criteria of International Standard ISO 10993-5:2009 [40], and the results of the study on mouse fibroblast L929 cells under the applied conditions. 

An additional layer of HA improved the hydrophilicity as well as antimicrobial and protein-repellent properties of the coatings, and even small surface changes of this type are major steps toward the advancement of anti-biofilm strategies. It is also important to mention that a detailed study of model PDMS films allows optimization and manipulation of surfaces towards desired properties regarding the coating and protein adsorption. In this way, it is possible to predict the behavior of real systems by studying model systems using QCM-D. Poor protein-repellent performance of the surface could predict poor biofilm properties and vice versa. Therefore, each material should be systematically studied as a model system and then transferred to a real system to obtain appropriate results.

## Figures and Tables

**Table 1:** QCM-D prepared samples and their abbreviations with descriptions.

Model Samples	
Au-PDMS	QSX301 coated with PDMS
Au-PDMS/Chi-77KS	Au-PDMS coated with Chi-77KS complex
Au-PDMS/Chi-77KS/AMOX	Au-PDMS coated with Chi-77KS/AMOX complex
Au-PDMS/Chi-77KS/HA	Au-PDMS coated with Chi-77KS complex and further by HA
Au-PDMS/Chi-77KS/AMOX/HA	Au-PDMS coated with Chi-77KS/AMOX complex and further by HA

**Table 2:** Real sample abbreviations with descriptions.

Real Samples	
PDMS	Reference PDMS disc
PDMS/Chi-77KS	PDMS disc coated with Chi-77KS complex
PDMS/Chi-77KS/AMOX	PDMS disc coated with Chi-77KS/AMOX complex
PDMS/Chi-77KS/HA	PDMS disc coated with Chi-77KS complex and further by HA
PDMS/Chi-77KS/AMOX/HA	PDMS disc coated with Chi-77KS/AMOX complex and further by HA

**Table 3:** Mix frequency change (Δ*f*), dissipation change (Δ*D*), desorption ratio Δ*f*_B_/Δ*f*_A_ and Δ*D*/Δ*f* ratio (A is Δ*f* and Δ*D* before rinsing, B is the final Δ*f* and Δ*D* after rinsing).

Mix	Δ*f* [Hz]		Δ*f*_B_/Δ*f*_A_ [%]	Δ*D* [10^−6^]		Δ*D*/Δ*f* [10^−7^ Hz^−1^]	
	A	B		A	B	A	B
Au-PDMS [35]	−126.2 ± 4.0	−104.7 ± 6.2	83.0	26.7 ± 2.1	20.1 ± 1.3	2.1	1.9
Au-PDMS/Chi-77KS [35]	−45.1 ± 3.4	−56.4 ± 3.3	125.1	8.3 ± 2.0	18.1 ± 1.1	1.8	3.2
Au-PDMS/Chi-77KS/AMOX [35]	−22.7 ± 2.2	−18.1 ± 1.4	79.7	4.8 ± 1.2	7.1 ± 0.9	2.1	3.9
Au-PDMS/Chi-77KS/HA	−34.8 ± 3.2	−29.9 ± 2.9	85.9	3.9 ± 1.9	3.6 ± 1.5	1.1	1.2
Au-PDMS/Chi-77KS/AMOX/HA	−21.3 ± 3.7	−19.1 ± 3.8	89.7	−6.4 ± 2.3	−7.4 ± 2.2	3.0	3.9

**Table 4:** Viscoelastic properties—the adsorbed mass (*Γ*_QCM_), film thickness (*h*_f_), viscosity (*η*_f_), and elastic shear modulus (*μ*_f_) of adsorbed mixed protein (Mix) layer onto various samples, including first (Chi-77KS, Chi-77KS/AMOX) and the second layer (HA).

Sample	*Γ*_QCM_ Wet [ng/m^2^]	*h*_f_ [nm]	*η*_f_ × 10^−3^ [Ns/m^2^]	*μ*_f_ × 10^4^ [N/m^2^]
Au-PDMS [35]	2662.60 ± 25.89	26.63 ± 2.86	0.002 ± 0.000	13.69 ± 1.52
Au-PDMS/Chi-77KS [35]	2550.40 ± 20.67	25.50 ± 1.62	0.002 ± 0.000	7.26 ± 0.86
Au-PDMS/Chi-77KS/AMOX [35]	2307.60 ± 18.66	23.08 ± 2.08	0.001 ± 0.000	1.76 ± 0.05
Au-PDMS/Chi-77KS/HA	800.31 ± 10.05	8.00 ± 0.54	0.003 ± 0.000	14.91 ± 0.91
Au-PDMS/Chi-77KS/AMOX/HA	341.61 ± 8.82	3.42 ± 0.12	0.715 ± 0.004	12.01 ± 1.35

**Table 5:** Biological reactivity assessment.

Sample		Cell Reactivity Grade		Biological Reactivity
		24 h	48 h	
NC		0	0	none
VC		0	0	none
PDMS% (*V*/*V*)	6.25	0	0	none
	12.5	0	0	none
	25	0	0	none
	50	0	0	none
	100	0	1	none/slight
PDMS/Chi-77KS/HA% (*V*/*V*)	6.25	0	0	none
	12.5	0	0	none
	25	0	0	none
	50	0	0	none
	100	0	1	none/slight
PDMS/Chi-77KS/AMOX/HA% (*V*/*V*)	6.25	0	0	none
	12.5	0	0	none
	25	0	0	none
	50	0–1	1	none-slight/slight
	100	0–1	2	none-slight/mild
PC		3	4	moderate/severe

NC—negative control, fresh growth media; VC—growth medium, incubated under extraction conditions; PC—positive control, etoposide (100 µg/mL).

## Data Availability

Not applicable.
